# Risk for Waterborne Transmission and Environmental Persistence of Avian Influenza Virus in a Wildlife/Domestic Interface in Mexico

**DOI:** 10.1007/s12560-024-09608-0

**Published:** 2024-07-21

**Authors:** Jessica Mateus-Anzola, Liliana Gaytan-Cruz, Ana Cecilia Espinosa-García, Beatriz Martínez-López, Rafael Ojeda-Flores

**Affiliations:** 1 Laboratorio de Ecología de Enfermedades y Una Salud, Departamento de Etología, Fauna Silvestre y Animales de Laboratorio, Facultad de Medicina Veterinaria y Zootecnia, Universidad Nacional Autónoma de México, Av. Universidad 3000, Edificio A, Delegación Coyoacán, Col. Ciudad Universitaria, 04510 Mexico City, Mexico; 2 Laboratorio Nacional de Ciencias de La Sostenibilidad, Instituto de Ecología, Universidad Nacional Autónoma de México, 04510 Mexico City, Mexico; 3 Center for Animal Disease Modeling and Surveillance, Department of Medicine and Epidemiology, School of Veterinary Medicine, University of California-Davis, Davis, CA 95616 USA

**Keywords:** Environmental exposure, Emerging infectious diseases, Epidemiologic factor, Multicriteria decision analysis, Disease transmission, Tenacity of influenza A virus

## Abstract

Aquatic habitats provide a bridge for influenza transmission among wild and domestic species. However, water sources pose highly variable physicochemical and ecological characteristics that affect avian influenza virus (AIV) stability. Therefore, the risk of survival or transmissibility of AIV in the environment is quite variable and has been understudied. In this study, we determine the risk of waterborne transmission and environmental persistence of AIV in a wild/domestic bird interface in the Central Mexico plateau (North America) during the winter season using a multi-criteria decision analysis (MCDA). A total of 13 eco-epidemiological factors were selected from public-access databases to develop the risk assessment. The MCDA showed that the Atarasquillo wetland presents a higher persistence risk in January. Likewise, most of the backyard poultry farms at this wild-domestic interface present a high persistence risk (50%). Our results suggest that drinking water may represent a more enabling environment for AIV persistence in contrast with wastewater. Moreover, almost all backyard poultry farms evidence a moderate or high risk of waterborne transmission especially farms close to water bodies. The wildlife/domestic bird interface on the Atarasquillo wetland holds eco-epidemiological factors such as the presence of farms in flood-prone areas, the poultry access to outdoor water, and the use of drinking-water troughs among multiple animal species that may enhance waterborne transmission of AIV. These findings highlight the relevance of understanding the influence of multiple factors on AIV ecology for early intervention and long-term control strategies.

**Supplementary Information:**

The online version contains supplementary material available at 10.1007/s12560-024-09608-0.

## Introduction

Environment plays a key role in viral transmission among infected and susceptible hosts (Keeler et al., 2014). Infected animals can introduce avian influenza viruses (AIVs) into aquatic habitats through fecal contamination and tracheal shedding (Nielsen et al., 2013). The AIVs contaminate open water bodies and become a source of infection or reinfection among wild and domestic avian populations (Keeler et al., 2012; Zhang et al., 2014).

Migratory waterfowl stopovers in proximity to backyard farms facilitate the emergence of new viral strains and enhance cross-species transmission (Müller-Theissen et al., 2022; Zhang et al., 2014). Multiple viral subtypes have been isolated from water mostly obtained in areas where farms overlap with wetlands (Das Gupta et al., 2022; Karasin et al., 2000; Mateus-Anzola et al., 2021). Therefore, household and free-range poultry on spatiotemporal coincidence with wild birds pose a high risk for influenza outbreaks (Hassan et al., 2020).

Environmental surveillance and laboratory-based studies have evidenced that IAVs may remain viable for extended periods outside of a biotic reservoir mainly in cold environmental conditions (Keeler et al., 2014; Ramey et al., 2022). Viral survival and stability depend on wild bird habitats’ physicochemical and ecological characteristics (Dalziel et al., 2016; Keeler et al., 2014; Tran et al., 2010). Likewise, viral transmission relies on farm epidemiological factors (Huang et al., 2016). However, there is limited data related to the influence of eco-epidemiological factors in the transmission and persistence of AIVs in environments shared between multiple species (Stallknecht et al., 2010). This study aims to fill some of those knowledge gaps by determining the risk of waterborne transmission and environmental persistence of AIVs at the wild/domestic bird interface using a multi-criteria decision analysis (MCDA). We illustrate our approach using data collected in Mexico during the winter season 2019–2020.

## Materials and Methods

### Study Area

The study area comprises one of the Lerma marshes, the Atarasquillo wetland, located in the Municipality of Lerma de Villada, State of Mexico, North America (19°13′–19°26′N, 99°22′–99°34′W). This Flora and Fauna Protection Area hosts great biological diversity, including endemic and threatened species such as the Mexican duck (*Anas diazi*) and twelve migratory wild bird species from North America (SEMARNAT-CONANP, 2018). Therefore, the Atarasquillo wetland is considered an ecosystem with high local and regional relevance for wild bird conservation in Mexico (Zepeda et al., 2014).

The Atarasquillo wetland represents a priority hydrological region based on productive and sociocultural activities. The region’s economy comprises artisanal fisheries, traditional hunting of waterfowl, farming, and grazing (Zepeda-Gómez et al., 2012). This Important Bird and Biodiversity Area (IBA) is surrounded by agricultural and livestock production systems mainly backyard poultry and pig farms with low bio-security measures that facilitate interspecies transmission (Gaytan-Cruz et al., 2020; Mateus-Anzola et al., 2020).

### Identification of Factors

Factors that influence the transmission and environmental persistence of AIVs were identified by a review of scientific literature. Three databases: Web of Science, PubMed, and Science Direct were searched for articles focused on the waterborne transmission of AIVs among wild birds and poultry, as well as the viral persistence in water. We used the search terms ((((influenza OR influenzavirus)) AND ((persistence OR survival OR stability OR viability OR inactivation OR tenacity OR survivability OR transmission OR infectivity OR infection OR infective OR infect)) AND ((surface water OR natural water OR wetland OR waterway OR watershed OR environmental water OR drinking water OR sewage OR wastewater)) AND ((factor)))). Studies in distilled water, peptone water, as well as human influenza viruses were excluded. Each factor was further evaluated through literature focusing on research articles with quantitative data and statistical significance on previous studies. A total of 13 eco-epidemiological factors were selected as inputs to develop risk assessments in a wild/domestic animal interface on the Atarasquillo wetland during the winter season (Table 1).

**Table 1:** Eco-epidemiological factors selected as model inputs, trends with AIV, reference values, and supporting citations

Factor	Trend with AIV	Reference values	References
Water temperature	Low temperatures keep the virus stable and infectious for longer	Optimal: <15 °C Suboptimal: 15–20 °C Non-optimal: >20 °C	(Domanska-Blicharz et al., 2010; Garamszegi & Møller, 2007; Keeler et al., 2012, 2014; Nazir et al., 2010; Nielsen et al., 2013; Perlas et al., 2023; Ramey et al., 2022; Shoham et al., 2012; Zhang et al., 2014)
Water salinity	Increased salinity has a detrimental effect on virus stability	Optimal: pond/freshwater (<0.5 ppt) Suboptimal: brackish (15 ppt) Non-optimal: seawater water (30 ppt)	(Bianchini et al., 2022; Domanska-Blicharz et al., 2010; Garamszegi & Møller, 2007; Hall et al., 2020; Keeler et al., 2012, 2014; Li, et al., 2011; Nielsen et al., 2013; Shoham et al., 2012)
Water pH	Neutral-to-basic pH facilitates long-term viral persistence and environmental stability	Optimal: neutral-slightly basic pH (7.0–8.5) Non-optimal: acid or basic pH (<7.0 or >8.5)	(Keeler et al., 2012, 2014; Ramey et al., 2022; Zhang et al., 2014)
Water ammonia concentration	High ammonia concentration is unfavorable for viral persistence	Optimal: <0.5 mg/L Non-optimal: ≥0.5 mg/L	(Keeler et al., 2014)
Water electrical conductivity	High viral persistence occurs on low electric conductivity	Optimal: low/moderate conductivity	(Nazir et al., 2010; Ramey et al., 2022)
Distance of natural or artificial water to farms	Open water in or bordering poultry farms increases the likelihood of concurrent use and viral transmission from wild birds to poultry	Optimal: <2 km Suboptimal: 2–10 km Non-optimal: >10 km	(Bouwstra et al., 2017; Cao et al., 2010; Dutta et al., 2022; Fang et al., 2008; Ferrer et al., 2014; Ge et al., 2012; Guerrini et al., 2014; Huang et al., 2016; Kjær et al., 2022; La Sala et al., 2019; Li et al., 2015; Liu et al., 2006; McDuie et al., 2019, 2022; Müller-Theissen et al., 2022; Paul et al., 2014; Shimizu et al., 2018; Si et al., 2013; Walsh et al., 2017; Wang et al., 2014)
Farm location	Areas covered by surface water are suitable for viral transmission and pose a higher risk of outbreak	Optimal: Farms located on flooded land or waterlogged areas	(Adhikari et al., 2009; Martin et al., 2011; Thanapongtharm et al., 2013; Van Boeckel et al., 2012)
Aquaculture farming	The use of ponds for fish culture can lead to a local increase in bird density and enhance viral transmission	Optimal: fish culture on farm ponds	(Pfeiffer et al., 2007)
Outdoor access areas	Poultry housed with access to outdoor water have a higher likelihood of spatial coincidence with wild birds leading to enhanced viral transmission	Optimal: Poultry have access to outdoor water	(Bouwstra et al., 2017; Desvaux et al., 2011; Dutta et al., 2022; Wang et al., 2014)
Drinking-water source	Drinking water supply from water bodies increases the risk of viral transmission	Optimal: surface water Non-optimal: tube-well water	(Das Gupta et al., 2022; Ferrer et al., 2014; Shafiq et al., 2021)
Drinking water-troughs	Healthy animals can be exposed to influenza virus through contaminated water	Optimal: shared among multiple species Suboptimal: shared only by one species Non-optimal: individual water-troughs	(Chen et al., 2023; Shafiq et al., 2021; Wang et al., 2017)
Drinking water quality	Water with feces is an efficient route of waterborne transmission	Optimal: drinking water contaminated with feces	(Claes et al., 2014; Himsworth et al., 2020)
Disposal of dead animals and wastage	Dumping dead birds or wastage near water bodies have a higher risk of outbreaks	Optimal: throw away nearby water bodies Suboptimal: buried on ground	(Dutta et al., 2022; Islam et al., 2022)

### Data Collection

**Fig. 1:**
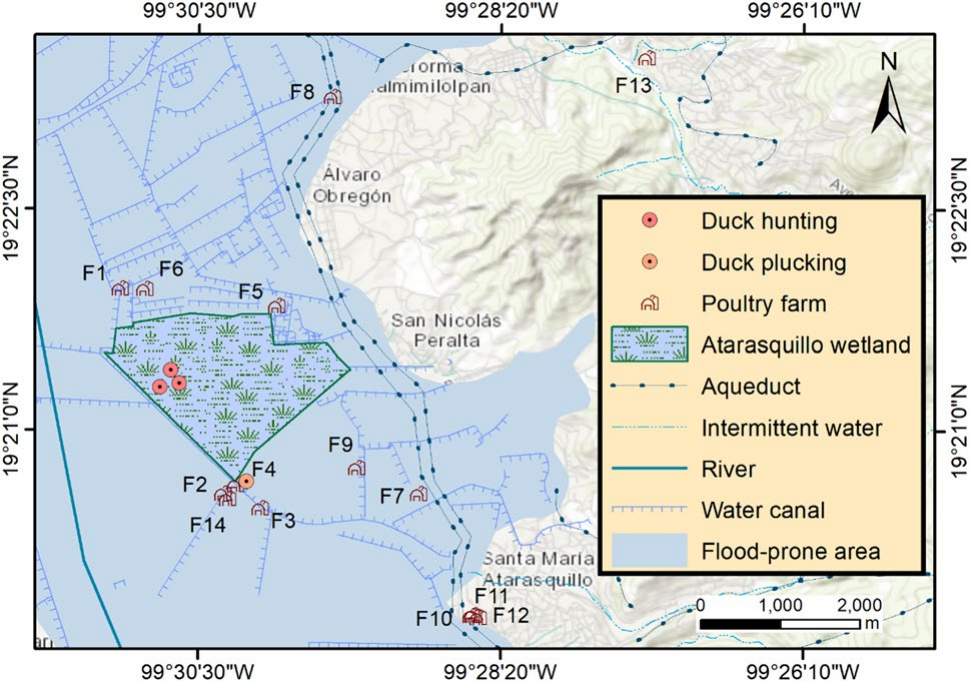
Geographical distribution of sampling sites in Lerma, State of Mexico. Map scale 1:70 000. ArcGis 10.8 (Color figure online)

The eco-epidemiological variables were collected from the Atarasquillo wetland and 14 backyard poultry farms during the winter season 2019–2020 using a convenience sampling method. Four sampling sites were considered within the Atarasquillo wetland: three sites where hunting duck activities were practiced, and one site where wild duck plucking was carried out on the shore of the wetland close to human settlements, crops, and poultry backyard farms (Fig. 1). The drinking water, wastewater, drainage ditch, and artificial pond were considered as sampling locations in the backyard poultry production systems.

Water temperature (°C) (HANNA, HI-98127 pocket meter) and water pH (HANNA, HI-98127 pH meter) were measured on-site twice in each sampling site. Water electrical conductivity (µS/cm), water ammonia concentration-NH3 (mg/L), and water salinity (psu) were measured in 500 ml water samples using a YSI 6600 Multi Parameter V2 Sonde. Farms were geo-located using a global positioning system (GPS) device and distances to the Atarasquillo wetland centroid (m) were computed using ArcGis 10.8 (CONANP, 2022; INEGI, 2022). Flood/waterlogged areas were identified according to soil susceptibility to flooding (i.e., vertisol and phaeozem were considered flooded soils) (Barragán & Figueroa, 2014). The soil types were explored with ArcGis 10.8 (INEGI, 2015; INIFAP-CONABIO, 2008). Data related to aquaculture farming, poultry outdoor access, drinking water source and quality, as well as disposal of dead animals and wastage were obtained by cross-sectional surveys.

### Weights Attribution for MCDA

Risk of environmental transmission and persistence of AIVs was determined by an MCDA approach similar to the one described by Zhao et al. (2022). The eco-epidemiological factors were considered as criteria and were weighted by the Mean Weight Method (Ezell et al., 2021; Odu, 2019), using the following equation:

$$Wj=\frac{1}{n}$$

where *Wj* is the criteria weight and *n* is the number of criteria.

The persistence MCDA model included five criteria (*Wj* = 0.2), while the transmission MCDA model accounted eight criteria (*Wj* = 0.125). The criteria were categorized in classes based on reference values. A weight from 0 to 3 was assigned to each class according to their suitability for AIV transmission or persistence in water (3 = high, 2 = mid, 1 = low, and 0 = none), where a higher value meant a higher likelihood of waterborne transmission or environmental persistence. The class weights (*Wi*) were normalized following the formula:

$$\text{Normalized} Wi =\frac{Wi-Wi \text{minimum}}{Wi \text{maximum}-Wi \text{minimum}}$$

The final weight was calculated by multiplying the normalized class weight with the criteria weight. The final score was the sum of the final weights obtained (Tables 2 and 3).

**Table 2:** Classes and weights of criteria in the influenza persistence MCDA

Criteria (*j*)	Class (*i*)	Class weight (*Wi*)^*a*^	Normalized class weight	Criteria weight (*Wj*)	Final weight
Water temperature	<15 °C	3	1.00	0.20	0.20
	15–20 °C	2	0.50		0.10
	>20 °C	1	0.00		0.00
Water salinity	<0.5 ppt	3	1.00	0.20	0.20
	0.5–30 ppt	2	0.50		0.10
	>30 ppt	1	0.00		0.00
Water pH	<7.0	1	0.00	0.20	0.00
	7.0–8.5	3	1.00		0.20
	>8.5	1	0.00		0.00
Water electrical conductivity	<200 µS/cm	3	1.00	0.20	0.20
	200–1000 µS/cm	2	0.50		0.10
	>1000 µS/cm	1	0.00		0.00
Water ammonia	<0.5 mg/L	3	1.00	0.20	0.20
	≥0.5 mg/L	1	0.00		0.00

^a^*Wi minimum* = 1*, Wi maximum* = 3

**Table 3:** Classes and weights of criteria in the influenza transmission MCDA

Criteria (*j*)	Class (*i*)	Class weight (*Wi*)^*a*^	Normalized class weight	Criteria weight (*Wj*)	Final weight
Distance from water bodies	<2000 m	3	1.00	0.13	0.13
	2000–5000 m	2	0.67		0.09
	5000–10000 m	1	0.33		0.04
Farm location	Flooded/waterlogged area	3	1.00	0.13	0.13
	Non-flooded land	0	0.00		0.00
Poultry outdoor access	Access to outdoor water	3	1.00	0.13	0.13
	Access to outdoor land	1	0.33		0.04
	No outdoor access	0	0.00		0.00
Drinking-water source	Surface water	3	1.00	0.13	0.13
	Tube-well water	1	0.33		0.04
Drinking water-troughs	Troughs for multiples species	3	1.00	0.13	0.13
	Troughs for the same species	2	0.67		0.09
	Individual water-troughs	1	0.33		0.04
Drinking water quality	Water with feces	3	1.00	0.13	0.13
	Water without feces	1	0.33		0.04
Disposal of dead animals and wastage	Throw away on water bodies	3	1.00	0.13	0.13
	Throw away/bury near water	1	0.33		0.04
	Bury, incinerate or compost	0	0.00		0.00
Aquaculture farming	Fish culture within the farm	3	1.00	0.13	0.13
	Pond available without fishes	1	0.33		0.04
	No pond within the farm	0	0.00		0.00

^a^*Wi minimum* = 0*, Wi maximum* = 3

### Waterborne Transmission and Environmental Persistence Risk

The final scores from the MCDA were classified into five risk categories: very low, low, moderate, high, and very high risk (Stenkamp-Strahm et al., 2020). The threshold was determined by dividing the maximum final score (1.00) into five levels (Table 4).

**Table 4:**
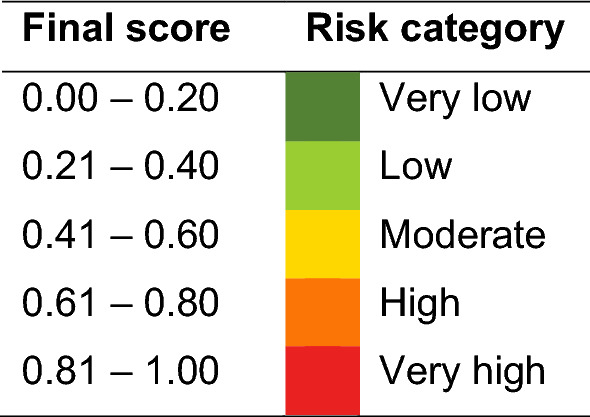
Final score and reclassification into risk categories (Color table online)

For example, if we have a sample with the following values: C1: 21.0 °C (temperature), C2: 0.6 ppt (salinity), C3: 7.6 (pH), C4: 867.5 µS/cm (electrical conductivity), and C5: 0.4 mg/L (ammonia), the sample would be a moderate risk according to the MCDA model (Table 5).

**Table 5:** Example of persistence MCDA model

Criteria (*j*)	Class (*i*)	Class weight (*Wi*)*	Normalized class weight	Criteria weight (*Wj*)	Final weight
C1	>20 °C	1	0.00	0.20	0.00
C2	0.5–30 ppt	2	0.50	0.20	0.10
C3	7.0–8.5	3	1.00	0.20	0.20
C4	200–1000 µS/cm	2	0.50	0.20	0.10
C5	<0.5 mg/L	3	1.00	0.20	0.20
				***Final score***	***0.60***

### Model Validation

A sensitivity analysis was performed to evaluate the stability of the outcomes under the uncertainty of the input variables**.** Although low pathogenic avian influenza viruses (LPAI) have been previously detected in resident and migratory wild bird species in the study area, no AIV has been reported in environmental samples of this wildlife/domestic bird interface (Gaytan-Cruz et al., 2020; Mateus-Anzola et al., 2020). Therefore, a one-at-a-time (OAT) method was conducted by varying one criterion at a time (Delgado & Sendra, 2004; Pianosi et al., 2016). The weight of one factor varied from 0 to 100% while the weights of the other criteria were adjusted to maintain the same percentage (e.g., an input weight of 60% on the variable factor represents weights of 10% to each of the other four criteria to sum 100%). Heat maps were used to represent the sensitivity analysis of the environmental persistence MCDAs (Online Resource 1 and 2) and the transmission persistence MCDA (Online Resource 3)**.**

## Results

### Environmental Persistence Risk

The average water temperature, salinity, pH, electrical conductivity, and ammonia on the Atarasquillo wetland and the water of backyard poultry farms during the winter season 2019–2020 is shown in Table 6. Physicochemical characteristics could not be obtained on farms F13 and F14.

**Table 6:** Physicochemical average values of water samples on a wildlife/domestic interface

Factor	Wetland	Drinking water	Wastewater/drainage	Pond
Temperature (°C)	15.9 ± 1.5	16.3 ± 3.9	16.7 ± 5.4	17.3 ± 0.6
Salinity (ppt)	0.3 ± 0.1	0.3 ± 0.3	0.9 ± 0.8	0.3 ± 0.1
pH	8.2 ± 0.02	8.2 ± 0.2	8.0 ± 0.6	7.2 ± 0.1
Conductivity (µS/cm)	501.1 ± 42.6	396.2 ± 355.0	1407.0 ± 1215.5	463.8 ± 182.8
Ammonia (mg/L)	0.04 ± 0.02	0.08 ± 0.12	2.7 ± 4.0	0.00

**Fig. 2:**
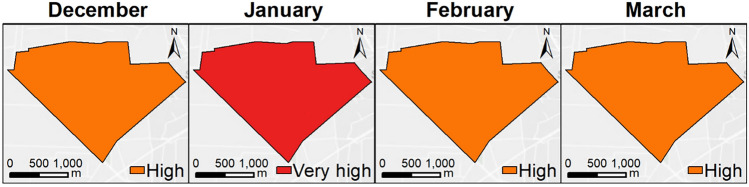
Persistence risk of AIVs in the Atarasquillo wetland during the winter season 2019–2020 using an MCDA model (Color figure online)

A high risk of environmental persistence was evidenced in December, February, and March, while a very high risk was evidenced in January, mainly between mid-January and early February on the Atarasquillo wetland (Fig. 2).

**Fig. 3:**
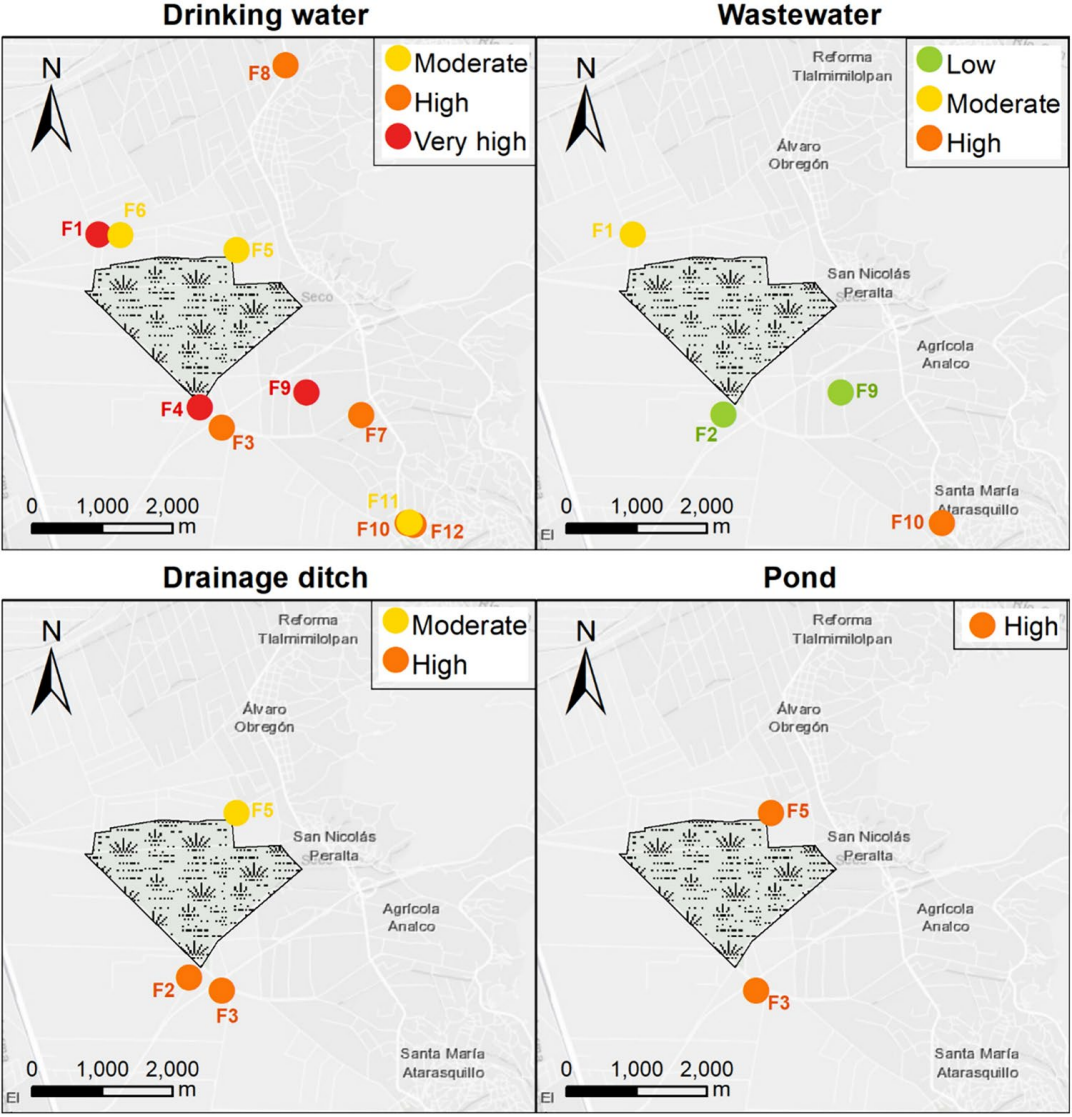
Persistence risk of AIVs in drinking water, wastewater, drainage ditch, and pond from poultry farms in a wildlife/domestic interface during the winter season 2019–2020 using an MCDA model (Color figure online)

In relation to backyard poultry farms, a moderate (27.27%), high (45.46%), and very high (27.27%) persistence risk was evidenced in drinking water. A moderate (33.33%) and high (66.67%) risk was presented in the drainage ditch. A low (50%), moderate (25%), and high (25%) risk was evidenced in wastewater, meanwhile, a high risk (100%) was observed in all the artificial ponds within the farms. Most of the farms presented a high persistence risk (50%) followed by a moderate risk (25%). Only two farms evidenced a low persistence risk (10%). None of the farms had a very low persistence risk (Fig. 3).

### Environmental Transmission Risk

Most of the backyard poultry farms were in flood-prone areas (71.42%) and almost 43% were located less than 2000 m from the Atarasquillo wetland’s centroid. Half of the poultry animals had access to outdoor water mainly to drainage ditches and artificial ponds. None of the animals have access to the Atarasquillo wetland. More than half of poultry (64.29%) share water troughs with another species and half of drinking water-throughs were dirty and contaminated with feces and feathers. Only three farms (21.42%) reported the disposal of poultry feces or eggs in/near water sources. None of the farmers mentioned the use of surface water as poultry drinking water.

**Fig. 4:**
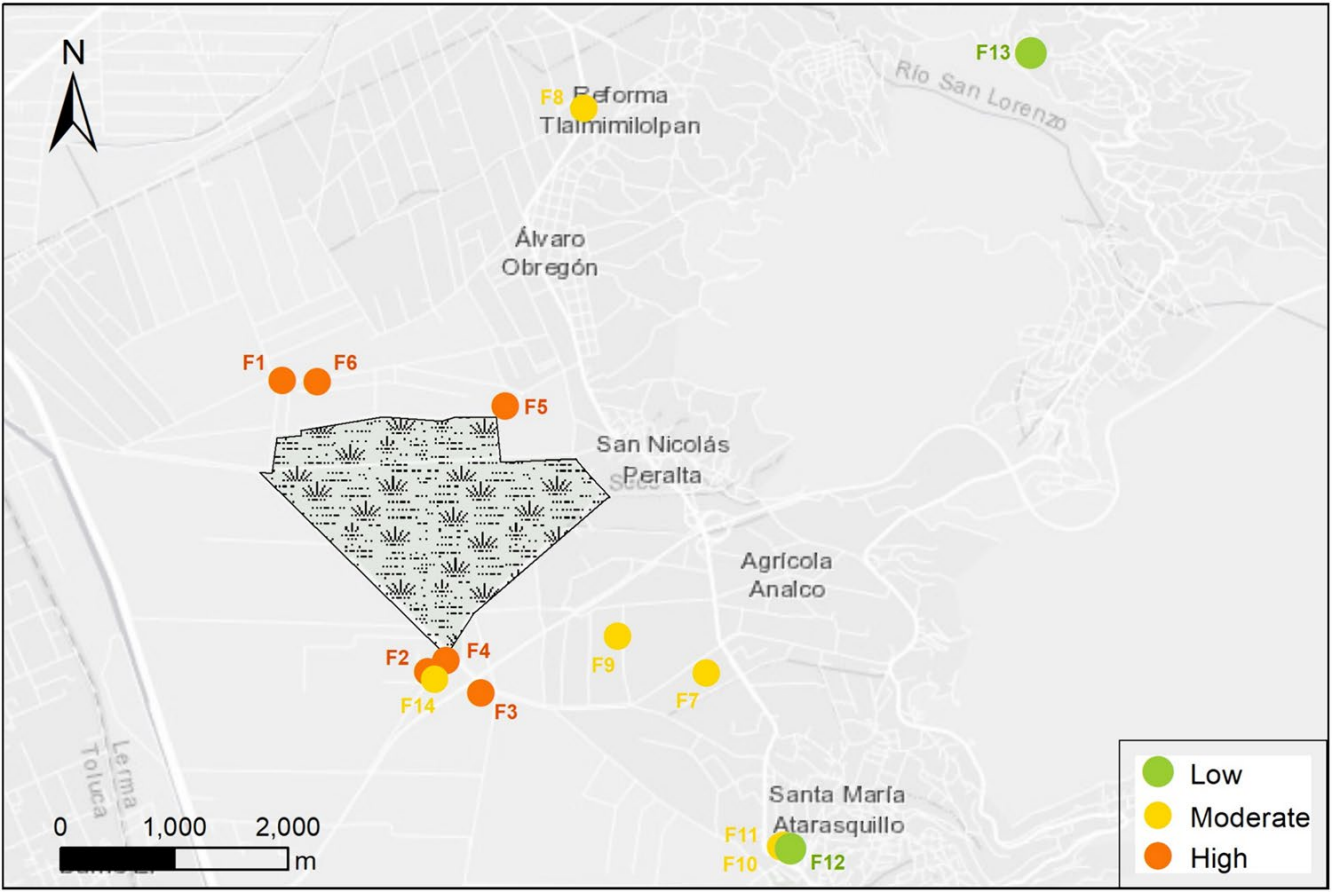
Waterborne transmission risk of AIVs in poultry farms of a wildlife/domestic interface during the winter season 2019–2020 using an MCDA model (Color figure online)

Almost all the backyard poultry farms had a moderate or high risk of waterborne transmission (85.72%), meanwhile, low risk was evidenced only in 14.28% of them. The backyard poultry systems with lower risk were located away from the Atarasquillo wetland (>4.3 km) (Fig. 4).

### Model Validation

The persistence risk on the Atarasquillo wetland was the same in the OAT analysis, in which a higher risk score was observed during January on the heat map. Likewise, the persistence risk and the transmission risk on backyard poultry farms were mostly comparable in the OAT analysis. A higher persistence risk score was evidenced in the drinking water, meanwhile, a lower risk score was evidenced in the wastewater on the heat map. Concerning the transmission risk, a higher risk was observed in six of the backyard poultry farms closest to the Atarasquillo wetland (F1 to F6) on the heat map (Online Resource 1, 2, and 3).

## Discussion

This study describes an MCDA approach to determine the risk of environmental persistence and waterborne transmission of AIV in a wild/domestic bird interface in central Mexico. The AIVs spread within wild waterbird populations may lead to viral contamination of natural habitats (Ahrens et al., 2022). During the winter season, a high density of migrating Anseriformes cohabit with resident species in the Atarasquillo wetland (Gaytan-Cruz et al., 2020; SEMARNAT-CONANP, 2018). One of the most remarkable results is that the coldest month evidences a higher persistence risk of AIV. Previous research has found a strong effect of temperature on environmental viability (Dalziel et al., 2016; Martin et al., 2018). Virus may remain infective for a few days at >20 °C, a few weeks at 10 °C, and for months at <0 °C in surface water (Nazir et al., 2010). Therefore, freshwater habitats could be a year-to-year reservoir of viruses to infect bird populations mainly in winter (Lang et al., 2008; Ramey et al., 2022).

Water sources represent a crucial environment in which infectious AIVs may reside outside of a biotic reservoir (Ramey et al., 2022). Recently, some mass mortality events in free-living mammal species such as the harbor seal are likely associated to environmental transmission of HPAI H5N1. Likewise, global HPAI outbreaks in poultry are possibly linked to indirect contact with wild birds (European Food Safety Authority et al., 2023). In our study, we did not attempt to record contact between household animals and wild birds. However, almost all the backyard poultry farms close to the Atarasquillo wetland evidenced a higher transmission risk. This outcome is in line with Si et al. (2013), who reported the occurrence of outbreaks mostly in areas where the location of farms or animal trade areas overlap with habitats for wild birds. Therefore, animal populations close to wetlands pose a high risk of influenza outbreaks (Hassan et al., 2020).

Shallow water bodies represent an AIV transmission medium for aquatic wild birds. Fecal matter, plumage, and oropharyngeal excretions with viral particles potentiate viral transmission efficacy in surface waters (Ahrens et al., 2022). In our study, artificial ponds within backyard poultry farms and channels of water evidenced a high risk of AIV persistence. Previous research has reported that a low viral titer suspended in the surface water is sufficient to start and set off an infection in wild ducks within a few days, mainly in a low volume of water. Small water bodies can hold moderate to high viral RNA loads for a long period due to a lower diluting effect on the virus available for infection compared to large water bodies (Ahrens et al., 2022).

A limited volume of accessible water may provide high viral titers and a long course of infection (Ahrens et al., 2022). According to Leung et al. (2007) poultry drinking water can provide higher isolation rates of the influenza virus than fecal droppings. Likewise, drinking water troughs may contain a great AIV subtype diversity (Mateus-Anzola et al., 2021). Interestingly, in our study, a very high-risk persistence score was found in some poultry farms’ drinking water. Experimental laboratory studies have reported AIV survivability of 8–48 h in drinking water troughs, as well as a viral concentrating effect. Nevertheless, survival time depends on the level of chlorination and the organic content of the water (Ahrens et al., 2022; Leung et al., 2007).

Effluents constitute an important factor in viral dissemination among poultry. Animal slurry (a liquid mixture of feces and urine added to litter, feed residues, washing water, and rainwater) contributes to AIV dissemination on poultry farms (Schmitz et al., 2020). Environmental samples collected from sewage may have high nucleic acid positivity rates of influenza (Bo et al., 2021; Guo et al., 2021). However, complex environments with high content of biological material (manure or feces) may retain infectivity for shorter periods than natural water (Schmitz et al., 2020). This is consistent with our findings where a low persistence risk was evidenced in wastewater.

The application of experimental results to field realities is complicated by the complexity and scale of these ecosystems (Stallknecht et al., 2010). Physicochemical properties such as temperature, pH, conductivity, ammonia concentration, and salinity can affect virus survival in different liquid environments (Keeler et al., 2014; Ramey et al., 2022; Schmitz et al., 2020). Nevertheless, other identified and unidentified factors prevailing in natural surface water may contribute to the effect of environmental persistence on AIV transmission dynamics among hosts (Martin et al., 2018; Nazir et al., 2010). The AIV subtype and its pathogenicity, bird density, UV light, and presence of biological compounds (freshwater crabs and microbial flora) were not evaluated in the MCDA. Likewise, further studies are required to assess the influence of viral, host, and biotic factors on AIV persistence and transmission in the wildlife-livestock interface.

Subtypes H1N1, H3N2, and H5N2 have been previously detected in wild birds in the study area (Gaytan-Cruz et al., 2020; Mateus-Anzola et al., 2020). However, no AIV has been detected in environmental samples at this wildlife/domestic bird interface. Negative samples may not reflect the true risk for AIV outbreaks (Belkhiria et al., 2018). Outbreaks of AIV in most tropical countries, such as Mexico, are mostly not detected due to limited surveillance infrastructure as well as the lack of standardization in sampling and reporting methods in both environmental and wild bird surveillance (Hood et al., 2020; Machalaba et al., 2015; Mateus-Anzola et al., 2021). Likewise, farmers from small-scale poultry farms usually do not report sick birds or unusual dead poultry to public health or agricultural authorities (Hall & Le, 2018; Hinjoy et al., 2023). This lack of detection and underreporting exacerbates the risk of unchecked AIV outbreaks in environments that enable the viral exchange between migratory waterfowl and domestic poultry.

In conclusion, the Atarasquillo wetland has eco-epidemiological factors that may enhance AIV survival and waterborne dissemination mainly in small-scale poultry farms close to the wetland. This MCDA provides valuable baseline information to identify the optimal environmental characteristics and high-risk epidemiological areas for AIV spreading as well as to develop early intervention strategies.

## Supplementary Information

Below is the link to the electronic supplementary material.

Supplementary file1 (PDF 107 KB)

Supplementary file2 (PDF 246 KB)

Supplementary file3 (PDF 259 KB)
