# Supplementary material for: Risk for Waterborne Transmission and Environmental Persistence of Avian Influenza Virus in a Wildlife/Domestic Interface in Mexico
Source: Food Environ Virol. 2024 Jul 21;16(4):458–69. doi: 10.1007/s12560-024-09608-0 (PMC11525396; doi:10.1007/s12560-024-09608-0)
Supplement: Supplementary file 1 — Supplementary file1 (PDF 107 KB) [file 12560_2024_9608_MOESM1_ESM.pdf]

Online Resource 1 Heat map showing the results of sensitivity analysis of environmental persistence on the Atarasquillo marsh

		0%	10%	20%	30%	40%	50%	60%	70%	80%	90%	100%
December	Tem	0.88	0.84	0.80	0.76	0.73	0.69	0.65	0.61	0.58	0.54	0.50
	Sal	0.75	0.78	0.80	0.83	0.85	0.88	0.90	0.93	0.95	0.98	1.00
	pH	0.75	0.78	0.80	0.83	0.85	0.88	0.90	0.93	0.95	0.98	1.00
	Amm	0.75	0.78	0.80	0.83	0.85	0.88	0.90	0.93	0.95	0.98	1.00
	Cond	0.88	0.84	0.80	0.76	0.73	0.69	0.65	0.61	0.58	0.54	0.50
January	Tem	0.88	0.89	0.90	0.91	0.93	0.94	0.95	0.96	0.98	0.99	1.00
	Sal	0.88	0.89	0.90	0.91	0.93	0.94	0.95	0.96	0.98	0.99	1.00
	pH	0.88	0.89	0.90	0.91	0.93	0.94	0.95	0.96	0.98	0.99	1.00
	Amm	0.88	0.89	0.90	0.91	0.93	0.94	0.95	0.96	0.98	0.99	1.00
	Cond	1.00	0.95	0.90	0.85	0.80	0.75	0.70	0.65	0.60	0.55	0.50
February	Tem	0.88	0.84	0.80	0.76	0.73	0.69	0.65	0.61	0.58	0.54	0.50
	Sal	0.75	0.78	0.80	0.83	0.85	0.88	0.90	0.93	0.95	0.98	1.00
	pH	0.75	0.78	0.80	0.83	0.85	0.88	0.90	0.93	0.95	0.98	1.00
	Amm	0.75	0.78	0.80	0.83	0.85	0.88	0.90	0.93	0.95	0.98	1.00
	Cond	0.88	0.84	0.80	0.76	0.73	0.69	0.65	0.61	0.58	0.54	0.50
March	Tem	0.88	0.84	0.80	0.76	0.73	0.69	0.65	0.61	0.58	0.54	0.50
	Sal	0.75	0.78	0.80	0.83	0.85	0.88	0.90	0.93	0.95	0.98	1.00
	pH	0.75	0.78	0.80	0.83	0.85	0.88	0.90	0.93	0.95	0.98	1.00
	Amm	0.75	0.78	0.80	0.83	0.85	0.88	0.90	0.93	0.95	0.98	1.00
	Cond	0.88	0.84	0.80	0.76	0.73	0.69	0.65	0.61	0.58	0.54	0.50

* Temp= Water temperature, Sal= Salinity, Amm= Ammonia concentration, Cond= Electrical conductivity.
