# Supplementary material for: Risk for Waterborne Transmission and Environmental Persistence of Avian Influenza Virus in a Wildlife/Domestic Interface in Mexico
Source: Food Environ Virol. 2024 Jul 21;16(4):458–69. doi: 10.1007/s12560-024-09608-0 (PMC11525396; doi:10.1007/s12560-024-09608-0)
Supplement: Supplementary file 2 — Supplementary file2 (PDF 246 KB) [file 12560_2024_9608_MOESM2_ESM.pdf]

Online Resource 2 Heat map showing the results of sensitivity analysis of environmental persistence on poultry farms.

**Drinking**		0%	10%	20%	30%	40%	50%	60%	70%	80%	90%	100%
F1	Tem	1.00	0.95	0.90	0.85	0.80	0.75	0.70	0.65	0.60	0.55	0.50
	Sal	0.88	0.89	0.90	0.91	0.93	0.94	0.95	0.96	0.98	0.99	1.00
	pH	0.88	0.89	0.90	0.91	0.93	0.94	0.95	0.96	0.98	0.99	1.00
	Amm	0.88	0.89	0.90	0.91	0.93	0.94	0.95	0.96	0.98	0.99	1.00
	Cond	0.88	0.89	0.90	0.91	0.93	0.94	0.95	0.96	0.98	0.99	1.00
F3	Tem	0.88	0.84	0.80	0.76	0.73	0.69	0.65	0.61	0.58	0.54	0.50
	Sal	0.75	0.78	0.80	0.83	0.85	0.88	0.90	0.93	0.95	0.98	1.00
	pH	0.75	0.78	0.80	0.83	0.85	0.88	0.90	0.93	0.95	0.98	1.00
	Amm	0.75	0.78	0.80	0.83	0.85	0.88	0.90	0.93	0.95	0.98	1.00
	Cond	0.88	0.84	0.80	0.76	0.73	0.69	0.65	0.61	0.58	0.54	0.50
F4	Tem	1.00	1.00	1.00	1.00	1.00	1.00	1.00	1.00	1.00	1.00	1.00
	Sal	1.00	1.00	1.00	1.00	1.00	1.00	1.00	1.00	1.00	1.00	1.00
	pH	1.00	1.00	1.00	1.00	1.00	1.00	1.00	1.00	1.00	1.00	1.00
	Amm	1.00	1.00	1.00	1.00	1.00	1.00	1.00	1.00	1.00	1.00	1.00
	Cond	1.00	1.00	1.00	1.00	1.00	1.00	1.00	1.00	1.00	1.00	1.00
F5	Tem	0.63	0.61	0.60	0.59	0.58	0.56	0.55	0.54	0.53	0.51	0.50
	Sal	0.50	0.55	0.60	0.65	0.70	0.75	0.80	0.85	0.90	0.95	1.00
	pH	0.75	0.68	0.60	0.53	0.45	0.38	0.30	0.23	0.15	0.08	0.00
	Amm	0.50	0.55	0.60	0.65	0.70	0.75	0.80	0.85	0.90	0.95	1.00
	Cond	0.63	0.61	0.60	0.59	0.58	0.56	0.55	0.54	0.53	0.51	0.50
F6	Tem	0.50	0.50	0.50	0.50	0.50	0.50	0.50	0.50	0.50	0.50	0.50
	Sal	0.38	0.44	0.50	0.56	0.63	0.69	0.75	0.81	0.88	0.94	1.00
	pH	0.63	0.56	0.50	0.44	0.38	0.31	0.25	0.19	0.13	0.06	0.00
	Amm	0.63	0.56	0.50	0.44	0.38	0.31	0.25	0.19	0.13	0.06	0.00
	Cond	0.38	0.44	0.50	0.56	0.63	0.69	0.75	0.81	0.88	0.94	1.00
F7	Tem	0.88	0.84	0.80	0.76	0.73	0.69	0.65	0.61	0.58	0.54	0.50
	Sal	0.75	0.78	0.80	0.83	0.85	0.88	0.90	0.93	0.95	0.98	1.00
	pH	0.75	0.78	0.80	0.83	0.85	0.88	0.90	0.93	0.95	0.98	1.00
	Amm	0.75	0.78	0.80	0.83	0.85	0.88	0.90	0.93	0.95	0.98	1.00
	Cond	0.88	0.84	0.80	0.76	0.73	0.69	0.65	0.61	0.58	0.54	0.50
F8	Tem	0.75	0.78	0.80	0.83	0.85	0.88	0.90	0.93	0.95	0.98	1.00
	Sal	0.88	0.84	0.80	0.76	0.73	0.69	0.65	0.61	0.58	0.54	0.50
	pH	0.75	0.78	0.80	0.83	0.85	0.88	0.90	0.93	0.95	0.98	1.00
	Amm	0.75	0.78	0.80	0.83	0.85	0.88	0.90	0.93	0.95	0.98	1.00
	Cond	0.88	0.84	0.80	0.76	0.73	0.69	0.65	0.61	0.58	0.54	0.50
F9	Tem	0.88	0.89	0.90	0.91	0.93	0.94	0.95	0.96	0.98	0.99	1.00
	Sal	0.88	0.89	0.90	0.91	0.93	0.94	0.95	0.96	0.98	0.99	1.00
	pH	0.88	0.89	0.90	0.91	0.93	0.94	0.95	0.96	0.98	0.99	1.00
	Amm	0.88	0.89	0.90	0.91	0.93	0.94	0.95	0.96	0.98	0.99	1.00

	Cond	1.00	0.95	0.90	0.85	0.80	0.75	0.70	0.65	0.60	0.55	0.50
F10	Tem	0.88	0.84	0.80	0.76	0.73	0.69	0.65	0.61	0.58	0.54	0.50
	Sal	0.75	0.78	0.80	0.83	0.85	0.88	0.90	0.93	0.95	0.98	1.00
	pH	0.75	0.78	0.80	0.83	0.85	0.88	0.90	0.93	0.95	0.98	1.00
	Amm	0.75	0.78	0.80	0.83	0.85	0.88	0.90	0.93	0.95	0.98	1.00
	Cond	0.88	0.84	0.80	0.76	0.73	0.69	0.65	0.61	0.58	0.54	0.50
F11	Tem	0.63	0.61	0.60	0.59	0.58	0.56	0.55	0.54	0.53	0.51	0.50
	Sal	0.50	0.55	0.60	0.65	0.70	0.75	0.80	0.85	0.90	0.95	1.00
	pH	0.75	0.68	0.60	0.53	0.45	0.38	0.30	0.23	0.15	0.08	0.00
	Amm	0.50	0.55	0.60	0.65	0.70	0.75	0.80	0.85	0.90	0.95	1.00
	Cond	0.63	0.61	0.60	0.59	0.58	0.56	0.55	0.54	0.53	0.51	0.50
F12	Tem	0.88	0.79	0.70	0.61	0.53	0.44	0.35	0.26	0.18	0.09	0.00
	Sal	0.63	0.66	0.70	0.74	0.78	0.81	0.85	0.89	0.93	0.96	1.00
	pH	0.63	0.66	0.70	0.74	0.78	0.81	0.85	0.89	0.93	0.96	1.00
	Amm	0.63	0.66	0.70	0.74	0.78	0.81	0.85	0.89	0.93	0.96	1.00
	Cond	0.75	0.73	0.70	0.68	0.65	0.63	0.60	0.58	0.55	0.53	0.50

Sewage

F1	Tem	0.75	0.68	0.60	0.53	0.45	0.38	0.30	0.23	0.15	0.08	0.00
	Sal	0.63	0.61	0.60	0.59	0.58	0.56	0.55	0.54	0.53	0.51	0.50
	pH	0.50	0.55	0.60	0.65	0.70	0.75	0.80	0.85	0.90	0.95	1.00
	Amm	0.50	0.55	0.60	0.65	0.70	0.75	0.80	0.85	0.90	0.95	1.00
	Cond	0.63	0.61	0.60	0.59	0.58	0.56	0.55	0.54	0.53	0.51	0.50
F2	Tem	0.75	0.68	0.60	0.53	0.45	0.38	0.30	0.23	0.15	0.08	0.00
	Sal	0.63	0.61	0.60	0.59	0.58	0.56	0.55	0.54	0.53	0.51	0.50
	pH	0.50	0.55	0.60	0.65	0.70	0.75	0.80	0.85	0.90	0.95	1.00
	Amm	0.50	0.55	0.60	0.65	0.70	0.75	0.80	0.85	0.90	0.95	1.00
	Cond	0.63	0.61	0.60	0.59	0.58	0.56	0.55	0.54	0.53	0.51	0.50
F9	Tem	0.13	0.21	0.30	0.39	0.48	0.56	0.65	0.74	0.83	0.91	1.00
	Sal	0.25	0.28	0.30	0.33	0.35	0.38	0.40	0.43	0.45	0.48	0.50
	pH	0.38	0.34	0.30	0.26	0.23	0.19	0.15	0.11	0.08	0.04	0.00
	Amm	0.38	0.34	0.30	0.26	0.23	0.19	0.15	0.11	0.08	0.04	0.00
	Cond	0.38	0.34	0.30	0.26	0.23	0.19	0.15	0.11	0.08	0.04	0.00
F10	Tem	0.88	0.79	0.70	0.61	0.53	0.44	0.35	0.26	0.18	0.09	0.00
	Sal	0.63	0.66	0.70	0.74	0.78	0.81	0.85	0.89	0.93	0.96	1.00
	pH	0.63	0.66	0.70	0.74	0.78	0.81	0.85	0.89	0.93	0.96	1.00
	Amm	0.63	0.66	0.70	0.74	0.78	0.81	0.85	0.89	0.93	0.96	1.00
	Cond	0.75	0.73	0.70	0.68	0.65	0.63	0.60	0.58	0.55	0.53	0.50

Drainage ditch

F2	Tem	0.63	0.66	0.70	0.74	0.78	0.81	0.85	0.89	0.93	0.96	1.00
	Sal	0.75	0.73	0.70	0.68	0.65	0.63	0.60	0.58	0.55	0.53	0.50
	pH	0.63	0.66	0.70	0.74	0.78	0.81	0.85	0.89	0.93	0.96	1.00
	Amm	0.63	0.66	0.70	0.74	0.78	0.81	0.85	0.89	0.93	0.96	1.00
	Cond	0.88	0.79	0.70	0.61	0.53	0.44	0.35	0.26	0.18	0.09	0.00

F3	Tem	0.88	0.84	0.80	0.76	0.73	0.69	0.65	0.61	0.58	0.54	0.50
	Sal	0.75	0.78	0.80	0.83	0.85	0.88	0.90	0.93	0.95	0.98	1.00
	pH	0.75	0.78	0.80	0.83	0.85	0.88	0.90	0.93	0.95	0.98	1.00
	Amm	0.75	0.78	0.80	0.83	0.85	0.88	0.90	0.93	0.95	0.98	1.00
	Cond	0.88	0.84	0.80	0.76	0.73	0.69	0.65	0.61	0.58	0.54	0.50
F5	Tem	0.75	0.68	0.60	0.53	0.45	0.38	0.30	0.23	0.15	0.08	0.00
	Sal	0.50	0.55	0.60	0.65	0.70	0.75	0.80	0.85	0.90	0.95	1.00
	pH	0.75	0.68	0.60	0.53	0.45	0.38	0.30	0.23	0.15	0.08	0.00
	Amm	0.50	0.55	0.60	0.65	0.70	0.75	0.80	0.85	0.90	0.95	1.00
	Cond	0.50	0.55	0.60	0.65	0.70	0.75	0.80	0.85	0.90	0.95	1.00
**Pond**												
F2	Tem	0.88	0.84	0.80	0.76	0.73	0.69	0.65	0.61	0.58	0.54	0.50
	Sal	0.75	0.78	0.80	0.83	0.85	0.88	0.90	0.93	0.95	0.98	1.00
	pH	0.75	0.78	0.80	0.83	0.85	0.88	0.90	0.93	0.95	0.98	1.00
	Amm	0.75	0.78	0.80	0.83	0.85	0.88	0.90	0.93	0.95	0.98	1.00
	Cond	0.88	0.84	0.80	0.76	0.73	0.69	0.65	0.61	0.58	0.54	0.50
F3	Tem	0.88	0.84	0.80	0.76	0.73	0.69	0.65	0.61	0.58	0.54	0.50
	Sal	0.75	0.78	0.80	0.83	0.85	0.88	0.90	0.93	0.95	0.98	1.00
	pH	0.75	0.78	0.80	0.83	0.85	0.88	0.90	0.93	0.95	0.98	1.00
	Amm	0.75	0.78	0.80	0.83	0.85	0.88	0.90	0.93	0.95	0.98	1.00
	Cond	0.88	0.84	0.80	0.76	0.73	0.69	0.65	0.61	0.58	0.54	0.50

* Temp= Water temperature, Sal= Salinity, Amm= Ammonia concentration, Cond= Electrical conductivity.
