# Supplementary material for: Risk for Waterborne Transmission and Environmental Persistence of Avian Influenza Virus in a Wildlife/Domestic Interface in Mexico
Source: Food Environ Virol. 2024 Jul 21;16(4):458–69. doi: 10.1007/s12560-024-09608-0 (PMC11525396; doi:10.1007/s12560-024-09608-0)
Supplement: Supplementary file 3 — Supplementary file3 (PDF 259 KB) [file 12560_2024_9608_MOESM3_ESM.pdf]

Online Resource 3. Heat map showing the results of sensitivity analysis of waterborne transmission.

		0%	10%	20%	30%	40%	50%	60%	70%	80%	90%	100%
F1	Dist	0.62	0.66	0.69	0.73	0.77	0.81	0.85	0.89	0.92	0.96	1.00
	Loc	0.62	0.66	0.69	0.73	0.77	0.81	0.85	0.89	0.92	0.96	1.00
	Aqu	0.76	0.69	0.61	0.53	0.46	0.38	0.30	0.23	0.15	0.08	0.00
	Out	0.62	0.66	0.69	0.73	0.77	0.81	0.85	0.89	0.92	0.96	1.00
	Sou	0.71	0.68	0.64	0.60	0.56	0.52	0.48	0.45	0.41	0.37	0.33
	Tro	0.62	0.66	0.69	0.73	0.77	0.81	0.85	0.89	0.92	0.96	1.00
	Qua	0.62	0.66	0.69	0.73	0.77	0.81	0.85	0.89	0.92	0.96	1.00
	Disp	0.76	0.69	0.61	0.53	0.46	0.38	0.30	0.23	0.15	0.08	0.00
F2	Dist	0.57	0.61	0.66	0.70	0.74	0.79	0.83	0.87	0.91	0.96	1.00
	Loc	0.57	0.61	0.66	0.70	0.74	0.79	0.83	0.87	0.91	0.96	1.00
	Aqu	0.71	0.64	0.57	0.50	0.43	0.36	0.29	0.21	0.14	0.07	0.00
	Out	0.57	0.61	0.66	0.70	0.74	0.79	0.83	0.87	0.91	0.96	1.00
	Sou	0.67	0.63	0.60	0.57	0.53	0.50	0.46	0.43	0.40	0.36	0.33
	Tro	0.57	0.61	0.66	0.70	0.74	0.79	0.83	0.87	0.91	0.96	1.00
	Qua	0.67	0.63	0.60	0.57	0.53	0.50	0.46	0.43	0.40	0.36	0.33
	Disp	0.67	0.63	0.60	0.57	0.53	0.50	0.46	0.43	0.40	0.36	0.33
F3	Dist	0.76	0.79	0.81	0.83	0.86	0.88	0.90	0.93	0.95	0.98	1.00
	Loc	0.76	0.79	0.81	0.83	0.86	0.88	0.90	0.93	0.95	0.98	1.00
	Aqu	0.76	0.79	0.81	0.83	0.86	0.88	0.90	0.93	0.95	0.98	1.00
	Out	0.76	0.79	0.81	0.83	0.86	0.88	0.90	0.93	0.95	0.98	1.00
	Sou	0.86	0.80	0.75	0.70	0.65	0.59	0.54	0.49	0.44	0.38	0.33
	Tro	0.76	0.79	0.81	0.83	0.86	0.88	0.90	0.93	0.95	0.98	1.00
	Qua	0.76	0.79	0.81	0.83	0.86	0.88	0.90	0.93	0.95	0.98	1.00
	Disp	0.90	0.81	0.72	0.63	0.54	0.45	0.36	0.27	0.18	0.09	0.00
F4	Dist	0.67	0.70	0.73	0.77	0.80	0.83	0.87	0.90	0.93	0.97	1.00
	Loc	0.67	0.70	0.73	0.77	0.80	0.83	0.87	0.90	0.93	0.97	1.00
	Aqu	0.81	0.73	0.65	0.57	0.49	0.40	0.32	0.24	0.16	0.08	0.00
	Out	0.67	0.70	0.73	0.77	0.80	0.83	0.87	0.90	0.93	0.97	1.00
	Sou	0.76	0.72	0.68	0.63	0.59	0.55	0.50	0.46	0.42	0.37	0.33
	Tro	0.67	0.70	0.73	0.77	0.80	0.83	0.87	0.90	0.93	0.97	1.00
	Qua	0.67	0.70	0.73	0.77	0.80	0.83	0.87	0.90	0.93	0.97	1.00
	Disp	0.76	0.72	0.68	0.63	0.59	0.55	0.50	0.46	0.42	0.37	0.33
F5	Dist	0.67	0.70	0.73	0.77	0.80	0.83	0.87	0.90	0.93	0.97	1.00
	Loc	0.67	0.70	0.73	0.77	0.80	0.83	0.87	0.90	0.93	0.97	1.00
	Aqu	0.67	0.70	0.73	0.77	0.80	0.83	0.87	0.90	0.93	0.97	1.00
	Out	0.67	0.70	0.73	0.77	0.80	0.83	0.87	0.90	0.93	0.97	1.00
	Sou	0.76	0.72	0.68	0.63	0.59	0.55	0.50	0.46	0.42	0.37	0.33
	Tro	0.67	0.70	0.73	0.77	0.80	0.83	0.87	0.90	0.93	0.97	1.00
	Qua	0.76	0.72	0.68	0.63	0.59	0.55	0.50	0.46	0.42	0.37	0.33

	Disp	0.81	0.73	0.65	0.57	0.49	0.40	0.32	0.24	0.16	0.08	0.00
F6	Dist	0.57	0.61	0.66	0.70	0.74	0.79	0.83	0.87	0.91	0.96	1.00
	Loc	0.57	0.61	0.66	0.70	0.74	0.79	0.83	0.87	0.91	0.96	1.00
	Aqu	0.71	0.64	0.57	0.50	0.43	0.36	0.29	0.21	0.14	0.07	0.00
	Out	0.57	0.61	0.66	0.70	0.74	0.79	0.83	0.87	0.91	0.96	1.00
	Sou	0.67	0.63	0.60	0.57	0.53	0.50	0.46	0.43	0.40	0.36	0.33
	Tro	0.62	0.62	0.63	0.63	0.64	0.64	0.65	0.65	0.66	0.66	0.67
	Qua	0.57	0.61	0.66	0.70	0.74	0.79	0.83	0.87	0.91	0.96	1.00
	Disp	0.71	0.64	0.57	0.50	0.43	0.36	0.29	0.21	0.14	0.07	0.00
F7	Dist	0.43	0.45	0.48	0.50	0.52	0.55	0.57	0.60	0.62	0.65	0.67
	Loc	0.38	0.44	0.50	0.57	0.63	0.69	0.75	0.81	0.88	0.94	1.00
	Aqu	0.52	0.47	0.42	0.37	0.31	0.26	0.21	0.16	0.10	0.05	0.00
	Out	0.48	0.46	0.45	0.43	0.42	0.40	0.39	0.37	0.36	0.34	0.33
	Sou	0.48	0.46	0.45	0.43	0.42	0.40	0.39	0.37	0.36	0.34	0.33
	Tro	0.38	0.44	0.50	0.57	0.63	0.69	0.75	0.81	0.88	0.94	1.00
	Qua	0.48	0.46	0.45	0.43	0.42	0.40	0.39	0.37	0.36	0.34	0.33
	Disp	0.52	0.47	0.42	0.37	0.31	0.26	0.21	0.16	0.10	0.05	0.00
F8	Dist	0.52	0.54	0.55	0.57	0.58	0.60	0.61	0.63	0.64	0.66	0.67
	Loc	0.48	0.53	0.58	0.63	0.69	0.74	0.79	0.84	0.90	0.95	1.00
	Aqu	0.62	0.56	0.49	0.43	0.37	0.31	0.25	0.19	0.12	0.06	0.00
	Out	0.62	0.56	0.49	0.43	0.37	0.31	0.25	0.19	0.12	0.06	0.00
	Sou	0.57	0.55	0.52	0.50	0.47	0.45	0.43	0.40	0.38	0.35	0.33
	Tro	0.48	0.53	0.58	0.63	0.69	0.74	0.79	0.84	0.90	0.95	1.00
	Qua	0.48	0.53	0.58	0.63	0.69	0.74	0.79	0.84	0.90	0.95	1.00
	Disp	0.57	0.55	0.52	0.50	0.47	0.45	0.43	0.40	0.38	0.35	0.33
F9	Dist	0.52	0.57	0.62	0.67	0.71	0.76	0.81	0.86	0.90	0.95	1.00
	Loc	0.52	0.57	0.62	0.67	0.71	0.76	0.81	0.86	0.90	0.95	1.00
	Aqu	0.67	0.60	0.53	0.47	0.40	0.33	0.27	0.20	0.13	0.07	0.00
	Out	0.62	0.59	0.56	0.53	0.50	0.47	0.45	0.42	0.39	0.36	0.33
	Sou	0.62	0.59	0.56	0.53	0.50	0.47	0.45	0.42	0.39	0.36	0.33
	Tro	0.52	0.57	0.62	0.67	0.71	0.76	0.81	0.86	0.90	0.95	1.00
	Qua	0.52	0.57	0.62	0.67	0.71	0.76	0.81	0.86	0.90	0.95	1.00
	Disp	0.67	0.60	0.53	0.47	0.40	0.33	0.27	0.20	0.13	0.07	0.00
F10	Dist	0.48	0.50	0.51	0.53	0.55	0.57	0.59	0.61	0.63	0.65	0.67
	Loc	0.57	0.51	0.46	0.40	0.34	0.29	0.23	0.17	0.11	0.06	0.00
	Aqu	0.57	0.51	0.46	0.40	0.34	0.29	0.23	0.17	0.11	0.06	0.00
	Out	0.43	0.49	0.54	0.60	0.66	0.71	0.77	0.83	0.89	0.94	1.00
	Sou	0.52	0.50	0.49	0.47	0.45	0.43	0.41	0.39	0.37	0.35	0.33
	Tro	0.43	0.49	0.54	0.60	0.66	0.71	0.77	0.83	0.89	0.94	1.00
	Qua	0.43	0.49	0.54	0.60	0.66	0.71	0.77	0.83	0.89	0.94	1.00
	Disp	0.57	0.51	0.46	0.40	0.34	0.29	0.23	0.17	0.11	0.06	0.00
F11	Dist	0.38	0.41	0.44	0.47	0.50	0.53	0.55	0.58	0.61	0.64	0.67
	Loc	0.48	0.43	0.38	0.33	0.29	0.24	0.19	0.14	0.10	0.05	0.00
	Aqu	0.48	0.43	0.38	0.33	0.29	0.24	0.19	0.14	0.10	0.05	0.00
	Out	0.43	0.42	0.41	0.40	0.39	0.38	0.37	0.36	0.35	0.34	0.33
	Sou	0.43	0.42	0.41	0.40	0.39	0.38	0.37	0.36	0.35	0.34	0.33

	Tro	0.38	0.41	0.44	0.47	0.50	0.53	0.55	0.58	0.61	0.64	0.67
	Qua	0.43	0.42	0.41	0.40	0.39	0.38	0.37	0.36	0.35	0.34	0.33
	Disp	0.33	0.40	0.47	0.53	0.60	0.67	0.73	0.80	0.87	0.93	1.00
F12	Dist	0.28	0.32	0.36	0.40	0.44	0.48	0.52	0.55	0.59	0.63	0.67
	Loc	0.38	0.34	0.30	0.27	0.23	0.19	0.15	0.11	0.08	0.04	0.00
	Aqu	0.38	0.34	0.30	0.27	0.23	0.19	0.15	0.11	0.08	0.04	0.00
	Out	0.38	0.34	0.30	0.27	0.23	0.19	0.15	0.11	0.08	0.04	0.00
	Sou	0.33	0.33	0.33	0.33	0.33	0.33	0.33	0.33	0.33	0.33	0.33
	Tro	0.33	0.33	0.33	0.33	0.33	0.33	0.33	0.33	0.33	0.33	0.33
	Qua	0.33	0.33	0.33	0.33	0.33	0.33	0.33	0.33	0.33	0.33	0.33
	Disp	0.24	0.31	0.39	0.47	0.54	0.62	0.69	0.77	0.85	0.92	1.00
F13	Dist	0.38	0.38	0.37	0.37	0.36	0.36	0.35	0.35	0.34	0.34	0.33
	Loc	0.43	0.38	0.34	0.30	0.26	0.21	0.17	0.13	0.09	0.04	0.00
	Aqu	0.43	0.38	0.34	0.30	0.26	0.21	0.17	0.13	0.09	0.04	0.00
	Out	0.38	0.38	0.37	0.37	0.36	0.36	0.35	0.35	0.34	0.34	0.33
	Sou	0.38	0.38	0.37	0.37	0.36	0.36	0.35	0.35	0.34	0.34	0.33
	Tro	0.33	0.37	0.40	0.43	0.47	0.50	0.53	0.57	0.60	0.64	0.67
	Qua	0.38	0.38	0.37	0.37	0.36	0.36	0.35	0.35	0.34	0.34	0.33
	Disp	0.28	0.36	0.43	0.50	0.57	0.64	0.71	0.79	0.86	0.93	1.00
F14	Dist	0.33	0.40	0.47	0.53	0.60	0.67	0.73	0.80	0.87	0.93	1.00
	Loc	0.33	0.40	0.47	0.53	0.60	0.67	0.73	0.80	0.87	0.93	1.00
	Aqu	0.48	0.43	0.38	0.33	0.29	0.24	0.19	0.14	0.10	0.05	0.00
	Out	0.48	0.43	0.38	0.33	0.29	0.24	0.19	0.14	0.10	0.05	0.00
	Sou	0.43	0.42	0.41	0.40	0.39	0.38	0.37	0.36	0.35	0.34	0.33
	Tro	0.38	0.41	0.44	0.47	0.50	0.53	0.55	0.58	0.61	0.64	0.67
	Qua	0.43	0.42	0.41	0.40	0.39	0.38	0.37	0.36	0.35	0.34	0.33
	Disp	0.48	0.43	0.38	0.33	0.29	0.24	0.19	0.14	0.10	0.05	0.00

* Dist = Distance of water bodies to farms, Loc= Farm location, Aqu= Aquaculture farming, Out= Outdoor access, Sou= Drinking-water source, Tro= Drinking water-troughs, Qua= Drinking water quality, Disp= Disposal of dead animals and wastage.
